# Supplementary material for: Parameter variations in personalized electrophysiological models of human heart ventricles
Source: PLoS One. 2021 Apr 28;16(4):e0249062. doi: 10.1371/journal.pone.0249062 (PMC8081243; doi:10.1371/journal.pone.0249062)
Supplement: S1 File — (PDF) [file pone.0249062.s001.pdf]

S1 Table. Data for Fig 1 in the main text. The distribution of the correlation coefficient (CC) and NRMSD between the simulated and measured ECG in BSM electrodes for every patient specific model.

**Correlation (CC)**	P1 (LV)	P1 (RV)	P2 (LV)	P2 (RV)	P3 (LV)	P3 (RV)	P4 (RV1)	P4 (RV2)	P5 (LV)	P6 (RV)
I	0.87	0.54	0.42	-0.80	0.88	-0.13	0.91	0.78	0.72	0.85
II	0.72	0.86	-0.34	0.87	0.49	0.95	0.91	0.34	0.87	0.86
III	0.32	0.84	0.35	0.88	0.78	0.97	0.90	-0.70	0.87	0.91
aVR	0.83	0.88	-0.56	0.85	0.55	0.93	0.91	0.70	0.88	0.76
aVL	0.83	0.80	0.63	0.88	0.86	0.68	0.26	-0.17	0.84	0.95
aVF	0.31	0.85	-0.04	0.87	0.66	0.96	0.91	-0.32	0.87	0.89
V1	0.92	0.03	-0.73	-0.79	0.90	0.86	0.94	0.85	0.74	0.82
V2	0.92	0.91	0.27	0.22	0.92	0.51	0.89	0.96	0.24	0.93
V3	0.30	0.91	0.57	0.91	0.93	0.61	0.40	0.72	0.88	0.94
V4	-0.31	0.91	0.39	0.91	0.82	0.92	0.82	-0.20	0.88	0.89
V5	-0.51	0.92	-0.13	0.88	0.33	0.98	0.93	0.63	0.88	0.64
V6	0.76	0.90	-0.25	0.85	0.67	0.80	0.95	0.90	0.86	0.66
**NRMSD**	P1 (LV)	P1 (RV)	P2 (LV)	P2 (RV)	P3 (LV)	P3 (RV)	P4 (RV1)	P4 (RV2)	P5 (LV)	P6 (RV)
I	11.53%	35.07%	41.20%	38.23%	31.55%	16.53%	13.42%	14.67%	20.32%	9.63%
II	15.66%	4.41%	8.78%	5.02%	41.59%	13.49%	14.53%	9.07%	6.06%	3.58%
III	19.11%	4.52%	7.24%	4.63%	24.97%	23.63%	22.78%	16.71%	6.23%	3.03%
aVR	14.55%	6.25%	15.08%	8.46%	36.86%	12.19%	15.60%	9.56%	8.46%	6.13%
aVL	12.43%	6.67%	9.06%	6.95%	28.30%	76.03%	47.56%	44.12%	9.38%	3.75%
aVF	22.39%	4.45%	7.95%	4.73%	30.42%	17.49%	17.67%	11.77%	6.12%	3.28%
V1	3.66%	7.54%	11.07%	17.51%	6.86%	7.51%	8.25%	6.33%	19.19%	5.17%
V2	3.07%	3.72%	8.46%	10.91%	8.04%	9.52%	5.15%	4.11%	18.36%	3.26%
V3	6.07%	3.68%	10.06%	6.91%	4.46%	17.99%	11.79%	11.97%	5.89%	3.77%
V4	7.59%	3.73%	15.42%	5.38%	4.36%	12.64%	17.58%	13.73%	5.98%	5.36%
V5	8.45%	3.70%	13.55%	6.42%	10.47%	5.57%	7.80%	6.97%	4.86%	8.30%
V6	19.55%	4.60%	15.71%	7.71%	19.51%	7.82%	6.73%	5.53%	7.04%	8.69%

S2 Table. Data for Fig 5 in the main text. Integrative effect of parameter variation in the physiological range on the model output signals (see annotation in the left column).

**Mean (RED,%)**	Endocard/epicard ratio	Apicobasal het. coefficient	Anisotropy ratio	Lungs conductivity	Blood conductivity	Liver conductivity	Spine conductivity
Transmembrane potential V_m in entire 3D myocardium	2.24	6.92	9.55	4.49	7.99	0.84	0.19
Transmembrane potential V_m on myocardium surface	1.73	7.01	9.45	5.14	8.20	0.96	0.22
Extracellular potential ϕ_e in entire 3D myocardium	10.61	12.52	32.68	28.36	36.59	7.20	1.25
Extracellular potential ϕ_e on myocardium surface	7.86	12.45	31.47	33.14	33.06	8.63	1.40
Electrical potential ϕ_b on torso surface	4.03	11.85	16.62	43.07	15.39	19.62	3.79
Electrical potential ϕ_i in electrodes	3.95	11.93	16.43	42.72	14.82	15.98	3.95
**Standart deviation**	Endocard/epicard ratio	Apicobasal het. coefficient	Anisotropy ratio	Lungs conductivity	Blood conductivity	Liver conductivity	Spine conductivity

Transmembrane potential V_m in entire 3D myocardium	1.61	0.12	2.19	3.27	1.02	1.32	0.25
Transmembrane potential V_m on myocardium surface	1.68	0.12	2.24	3.60	1.05	1.45	0.27
Extracellular potential ϕ_e in entire 3D myocardium	8.17	0.80	2.32	11.84	8.29	4.97	1.34
Extracellular potential ϕ_e on myocardium surface	6.72	0.78	1.99	11.34	8.40	5.56	1.42
Electrical potential ϕ_b on torso surface	1.22	1.36	3.04	17.62	4.56	6.89	2.26
Electrical potential ϕ_i in electrodes	1.27	1.32	3.07	15.49	4.17	4.74	2.17

S3 Table. Data for Fig 6 in the main text. Variation in the temporal characteristics of depolarisation and repolarisation (see annotation on the top of panels) in the models under univariable parameter variation (see annotation in the left column).

	**Late activation time (% of reference)**													
	Endocard/epicard ratio		Apicobasal het. coefficient		Anisotropy ratio		Lungs conductivity		Blood conductivity		Liver conductivity		Spine conductivity	
P1 (LV)	100.00	100.00	100.00	100.00	93.93	103.45	99.36	99.86	98.78	102.09	100.00	100.00	100.00	100.00
P1 (RV)	99.92	100.05	100.00	100.00	95.13	103.37	99.22	99.83	98.48	102.60	100.00	100.00	99.24	100.02
P3 (RV)	99.90	100.07	100.00	100.00	94.01	104.76	99.11	99.81	98.25	102.28	100.00	100.00	100.00	100.00
P3 (LV)	99.95	100.02	100.00	100.00	94.58	104.26	99.40	99.87	98.24	102.05	100.00	100.00	100.00	100.00
P2 (LV)	99.93	100.02	100.00	100.00	96.67	102.53	100.00	100.00	97.81	103.76	100.00	100.00	100.00	100.00
P2 (RV)	99.85	100.74	100.00	100.00	93.87	105.11	99.99	100.01	98.53	102.52	100.00	100.00	100.00	100.00
P4 (RV1)	99.93	100.05	100.00	100.00	92.13	105.60	97.29	99.71	99.34	100.58	100.00	100.00	100.00	100.00
P4 (RV2)	99.94	100.02	100.00	100.00	94.30	104.09	95.88	99.42	99.33	101.15	100.00	100.00	100.00	100.00
P5 (LV)	99.61	100.15	100.00	100.00	97.13	102.31	99.06	99.80	97.22	103.22	99.07	100.38	100.00	100.00
P6 (RV)	99.89	100.08	100.00	100.00	94.74	103.87	99.03	99.80	98.10	102.47	100.00	100.00	100.00	100.00
	**APD dispersion (% of reference)**													
	Endocard/epicard ratio		Apicobasal het. coefficient		Anisotropy ratio		Lungs conductivity		Blood conductivity		Liver conductivity		Spine conductivity	
P1 (LV)	96.31	98.66	77.26	100.00	100.98	102.11	100.00	100.00	99.78	101.46	100.00	100.00	100.00	100.00
P1 (RV)	99.27	100.21	76.44	100.00	99.73	100.08	100.00	100.00	100.00	100.00	98.18	100.75	100.00	100.00
P3 (RV)	102.07	104.26	77.22	100.00	96.24	98.61	99.78	103.08	100.00	102.34	100.00	100.00	100.00	100.00
P3 (LV)	101.77	102.13	79.17	100.00	96.37	99.24	98.05	99.61	100.00	100.00	100.00	100.00	100.00	100.00

P2 (LV)	100.00	101.97	77.38	100.00	101.44	102.11	100.00	100.00	98.40	100.24	100.00	100.00	100.00	100.00
P2 (RV)	101.29	102.13	76.23	100.00	99.30	100.03	99.96	101.55	100.00	102.21	100.00	100.00	100.00	100.00
P4 (RV1)	97.97	99.48	79.76	100.00	101.99	102.04	100.00	100.00	99.75	101.63	100.00	100.00	100.00	100.00
P4 (RV2)	100.00	102.35	79.50	100.00	101.83	103.06	100.00	100.00	100.00	100.00	100.00	100.00	100.00	100.00
P5 (LV)	99.84	102.21	75.13	100.00	98.08	98.69	100.00	100.00	99.77	101.51	100.00	100.00	100.00	100.00
P6 (RV)	94.11	97.15	78.50	100.00	97.71	98.53	100.00	100.00	100.00	100.00	100.00	100.00	100.00	100.00
	**Repolarization dispersion (% of reference)**													
	Endocard/epicard ratio		Apicobasal het. coefficient		Anisotropy ratio		Lungs conductivity		Blood conductivity		Liver conductivity		Spine conductivity	
P1 (LV)	100.45	100.82	100.00	100.51	82.71	92.10	101.36	102.91	100.00	104.09	100.00	100.00	100.00	100.00
P1 (RV)	100.00	100.00	100.00	103.27	81.86	91.74	101.97	104.20	100.00	105.60	100.00	100.00	100.00	100.00
P3 (RV)	91.43	92.69	100.00	104.20	69.37	81.10	99.98	101.00	100.00	104.49	100.00	100.00	100.00	100.00
P3 (LV)	99.93	100.48	100.00	100.24	80.78	89.49	98.85	99.36	100.00	104.15	100.00	100.00	100.00	100.00
P2 (LV)	94.99	95.90	100.00	101.24	86.34	91.85	100.86	100.89	100.00	106.52	99.64	100.86	100.00	100.00
P2 (RV)	96.81	98.23	100.00	102.75	74.95	85.49	99.89	101.53	100.00	106.82	100.00	100.00	100.00	100.00
P4 (RV1)	95.16	96.46	98.84	100.00	80.13	90.90	102.07	104.75	100.00	101.54	100.00	100.00	100.00	100.00
P4 (RV2)	93.88	95.15	100.00	101.37	80.65	90.22	102.44	108.27	99.80	101.35	100.00	100.00	100.00	100.00
P5 (LV)	93.65	94.32	100.00	110.26	78.95	86.43	100.00	100.00	100.00	109.05	100.00	100.00	100.00	100.00
P6 (RV)	96.09	96.87	94.62	100.00	88.75	92.72	99.98	100.74	100.00	103.59	100.00	100.00	100.00	100.00

S4 Table. Data for Fig 7 in the main text. Variation in the amplitude characteristics of depolarisation and repolarisation (see annotation on the top of panels) due to univariable parameter variations (see annotation in the left columns) in the models.

	**EP max. activation amplitude (% of reference)**													
	Endocard/epicard ratio		Apicobasal het. coefficient		Anisotropy ratio		Lungs conductivity		Blood conductivity		Liver conductivity		Spine conductivity	
P1 (LV)	99.51	100.16	100.00	100.00	96.56	102.43	71.43	96.10	99.51	100.98	99.99	100.00	100.00	100.01
P1 (RV)	99.83	100.35	100.00	100.03	94.77	105.37	83.19	98.23	99.70	100.29	99.95	100.00	99.89	100.03
P3 (RV)	99.85	100.35	100.00	100.02	96.11	103.77	78.85	97.33	98.72	101.86	100.03	100.06	99.93	100.10
P3 (LV)	99.45	100.16	100.00	100.01	95.54	102.33	72.32	96.27	99.22	101.31	99.99	100.00	99.65	100.33
P2 (LV)	99.81	100.26	99.98	100.00	94.99	104.45	72.42	96.73	96.86	105.35	98.87	103.47	100.00	100.00
P2 (RV)	99.04	100.36	100.00	100.00	96.37	102.05	80.84	97.47	97.29	103.25	100.05	100.62	99.96	100.07
P4 (RV1)	99.71	100.03	100.00	100.06	95.99	102.59	63.64	95.36	99.96	100.21	99.94	99.99	100.00	100.04
P4 (RV2)	99.58	100.06	100.00	100.04	96.98	102.46	75.09	97.42	99.40	101.93	100.08	100.26	99.99	100.05
P5 (LV)	99.88	100.25	100.00	100.00	95.77	103.73	81.18	97.54	98.74	102.95	99.95	100.09	100.00	100.00
P6 (RV)	99.31	100.01	100.00	100.03	93.10	103.40	73.56	95.75	99.57	100.00	99.99	99.99	99.96	100.04
	**EP max. recovery amplitude (% of reference)**													

	Endocard/epicard ratio		Apicobasal het. coefficient		Anisotropy ratio		Lungs conductivity		Blood conductivity		Liver conductivity		Spine conductivity	
P1 (LV)	98.89	100.25	99.98	100.00	91.47	105.65	65.71	95.83	97.68	103.85	99.91	99.99	99.97	100.01
P1 (RV)	98.86	100.30	100.00	100.78	98.30	100.36	99.36	99.94	97.87	103.12	100.11	103.94	99.12	100.40
P3 (RV)	99.72	101.33	99.67	100.02	92.31	106.11	91.58	98.22	95.08	108.26	100.07	100.32	99.90	100.08
P3 (LV)	99.05	100.08	99.98	100.02	93.09	105.36	66.99	95.90	96.56	105.62	99.98	100.00	99.99	100.01
P2 (LV)	98.25	100.24	100.00	100.63	92.51	106.09	69.17	96.35	95.10	109.33	99.91	99.99	100.00	100.00
P2 (RV)	99.77	100.61	99.70	100.61	98.54	100.57	99.80	99.99	96.32	105.99	100.22	104.10	99.98	100.02
P4 (RV1)	99.96	100.27	100.00	100.76	95.19	102.98	54.89	92.69	98.95	100.63	99.95	100.00	99.98	100.06
P4 (RV2)	99.98	101.13	100.00	101.90	94.33	104.06	78.12	96.64	96.68	105.82	100.06	100.60	99.95	100.14
P5 (LV)	99.52	102.30	99.90	104.09	95.02	106.75	90.38	98.66	87.72	120.65	100.15	100.88	99.99	100.02
P6 (RV)	98.57	100.12	89.17	100.00	96.03	106.10	90.73	98.86	98.83	105.99	99.97	100.05	99.89	100.06
	**Max. absolute QRS amplitude (% of reference)**													
	Endocard/epicard ratio		Apicobasal het. coefficient		Anisotropy ratio		Lungs conductivity		Blood conductivity		Liver conductivity		Spine conductivity	
P1 (LV)	99.16	100.21	100.00	100.08	92.04	105.47	82.93	98.20	98.37	100.38	99.89	100.65	99.55	100.28
P1 (RV)	99.48	100.35	100.00	100.01	99.80	100.90	91.48	98.87	90.05	114.04	100.36	101.81	99.53	100.20
P3 (RV)	99.32	100.48	100.00	100.01	94.82	105.81	93.74	98.83	89.72	113.23	100.75	105.55	99.43	100.47
P3 (LV)	99.17	100.01	100.00	100.09	96.88	102.53	91.56	99.25	99.10	100.01	100.41	102.74	99.23	100.30
P2 (LV)	99.71	100.03	100.00	100.05	97.64	100.59	88.06	98.57	99.07	100.80	84.35	97.24	99.70	100.32
P2 (RV)	99.92	101.56	100.00	100.02	99.31	102.35	85.74	98.18	89.45	115.85	100.55	107.64	99.87	100.07
P4 (RV1)	99.88	100.01	100.00	100.06	90.67	113.92	102.68	111.25	96.73	105.77	98.01	99.33	98.96	100.57
P4 (RV2)	99.38	100.20	99.99	100.00	95.52	105.27	95.02	99.61	93.64	108.52	101.14	109.49	99.93	100.06
P5 (LV)	99.14	100.72	100.00	100.00	93.40	109.85	97.97	100.91	82.47	125.77	100.88	116.04	100.00	100.12
P6 (RV)	99.71	100.03	100.00	100.03	94.90	115.69	96.79	99.48	97.57	108.17	100.08	102.04	99.54	100.25
	**Max. absolute T-wave amplitude (% of reference)**													
	Endocard/epicard ratio		Apicobasal het. coefficient		Anisotropy ratio		Lungs conductivity		Blood conductivity		Liver conductivity		Spine conductivity	
P1 (LV)	98.58	102.00	96.55	100.00	93.72	100.80	76.76	97.72	99.51	101.58	100.11	102.49	99.89	100.07
P1 (RV)	98.35	101.50	100.00	109.66	94.61	103.95	93.12	99.14	94.06	110.25	100.15	100.71	99.88	100.11
P3 (RV)	98.32	101.65	100.00	109.08	98.85	100.13	87.38	98.26	93.15	110.62	101.12	105.19	98.62	101.90
P3 (LV)	98.63	100.61	94.60	100.00	99.39	100.00	92.45	99.60	96.92	104.81	101.02	103.63	99.50	100.71
P2 (LV)	98.32	102.07	95.75	100.00	90.03	107.00	87.20	98.47	97.49	103.86	91.84	98.83	99.45	100.27
P2 (RV)	99.52	103.65	100.00	111.80	97.78	104.48	88.88	98.58	89.40	117.42	100.70	108.28	99.85	100.06
P4 (RV1)	99.24	101.40	89.56	100.00	98.38	101.18	101.80	106.54	95.90	106.13	100.28	108.54	98.90	100.59
P4 (RV2)	99.61	101.26	100.00	110.13	98.07	100.31	87.91	98.71	92.82	110.16	101.42	104.94	99.87	100.11
P5 (LV)	97.84	100.54	100.00	143.65	99.76	102.30	102.20	110.31	84.37	122.71	100.31	102.65	99.76	100.12
P6 (RV)	98.64	102.94	81.60	100.00	96.43	108.31	83.93	97.92	93.74	110.18	100.16	103.81	99.13	100.44

S5 Table. Data for Fig 4 in the main text. Dependence of the integrative distance (RED) between simulated and patient ECG upon each varied model parameter in the patient specific models.

**Endo./epi. coefficient**					
Param. values	0	0.25	0.5	0.75	1
P1 (LV)	85.16	78.57	79.25	80.49	85.16
P6 (RV)	79.30	70.11	70.34	70.96	79.62
P3 (LV)	70.15	65.81	66.44	67.23	72.43
P5 (LV)	141.43	121.31	121.59	122.55	142.83
P4 (RV1)	161.14	151.22	150.61	150.61	162.12
P1 (RV)	80.87	80.86	81.45	82.06	80.87
P4 (RV2)	150.28	155.71	155.48	155.17	154.87

P@ (RV)	153.96	147.64	150.04	151.48	159.67
P2 (LV)	338.09	314.98	316.73	319.25	340.40
P3 (RV)	169.03	167.01	166.91	167.62	173.33
**Apicobasal heterogeneity coefficient**					
Param. values	0	0.5	1		
P1 (LV)	85.11	79.03	79.25		
P6 (RV)	64.38	65.15	70.34		
P3 (LV)	70.94	64.69	66.44		
P5 (LV)	125.83	118.83	121.59		
P4 (RV1)	129.62	141.06	150.61		
P1 (RV)	81.72	79.42	81.45		
P4 (RV2)	178.42	163.55	155.48		
P@ (RV)	157.97	151.97	150.04		
P2 (LV)	320.12	315.86	316.73		
P3 (RV)	182.27	169.93	166.91		
**Anisotropy ratio**					
Param. values	1.6	2.5	4	6	
P1 (LV)	89.50	79.25	80.18	89.65	
P6 (RV)	77.36	70.34	74.88	77.73	
P3 (LV)	75.51	66.44	62.48	60.14	
P5 (LV)	111.08	121.59	126.24	125.60	
P4 (RV1)	162.14	150.61	146.25	142.09	
P1 (RV)	83.16	81.45	84.36	87.03	
P4 (RV2)	169.35	155.48	149.42	146.10	
P@ (RV)	143.37	150.04	140.85	130.00	
P2 (LV)	255.71	316.73	349.22	357.13	
P3 (RV)	173.43	166.91	161.15	157.74	
**Lungs conductivity**					
Param. values	0.3	1	1.3		
P1 (LV)	79.25	70.61	76.16		
P6 (RV)	70.34	67.74	68.29		
P3 (LV)	66.44	70.19	76.47		
P5 (LV)	121.59	145.13	147.79		
P4 (RV1)	150.61	160.40	155.83		
P1 (RV)	81.45	88.26	90.23		
P4 (RV2)	155.48	158.79	157.45		
P@ (RV)	150.04	153.15	153.07		
P2 (LV)	316.73	253.44	240.92		
P3 (RV)	166.91	216.71	226.96		
**Blood conductivity**					
Param. values	4	7	10		
P1 (LV)	83.31	79.25	77.11		
P6 (RV)	73.40	70.34	69.39		

P3 (LV)	69.24	66.44	65.57		
P5 (LV)	129.05	121.59	118.82		
P4 (RV1)	165.31	150.61	143.72		
P1 (RV)	87.73	81.45	78.92		
P4 (RV2)	178.71	155.48	141.62		
P@ (RV)	156.16	150.04	145.42		
P2 (LV)	327.27	316.73	308.63		
P3 (RV)	184.67	166.91	158.72		
**Liver conductivity**					
Param. values	0.277	1	2.7		
P1 (LV)	73.04	74.85	79.25		
P6 (RV)	67.82	68.42	70.34		
P3 (LV)	64.31	64.26	66.44		
P5 (LV)	111.72	115.73	121.59		
P4 (RV1)	155.51	150.91	150.61		
P1 (RV)	82.97	81.90	81.45		
P4 (RV2)	160.82	157.53	155.48		
P@ (RV)	161.43	157.49	150.04		
P2 (LV)	287.48	299.82	316.73		
P3 (RV)	177.69	173.10	166.91		
**Spine conductivity**					
Param. values	0.05	0.2	0.6		
P1 (LV)	80.01	79.25	78.22		
P6 (RV)	70.74	70.34	69.83		
P3 (LV)	67.03	66.44	65.50		
P5 (LV)	120.60	121.59	123.43		
P4 (RV1)	151.08	150.61	150.20		
P1 (RV)	81.18	81.45	82.40		
P4 (RV2)	156.38	155.48	155.30		
P@ (RV)	147.18	150.04	155.29		
P2 (LV)	316.60	316.73	317.18		
P3 (RV)	164.39	166.91	170.89		

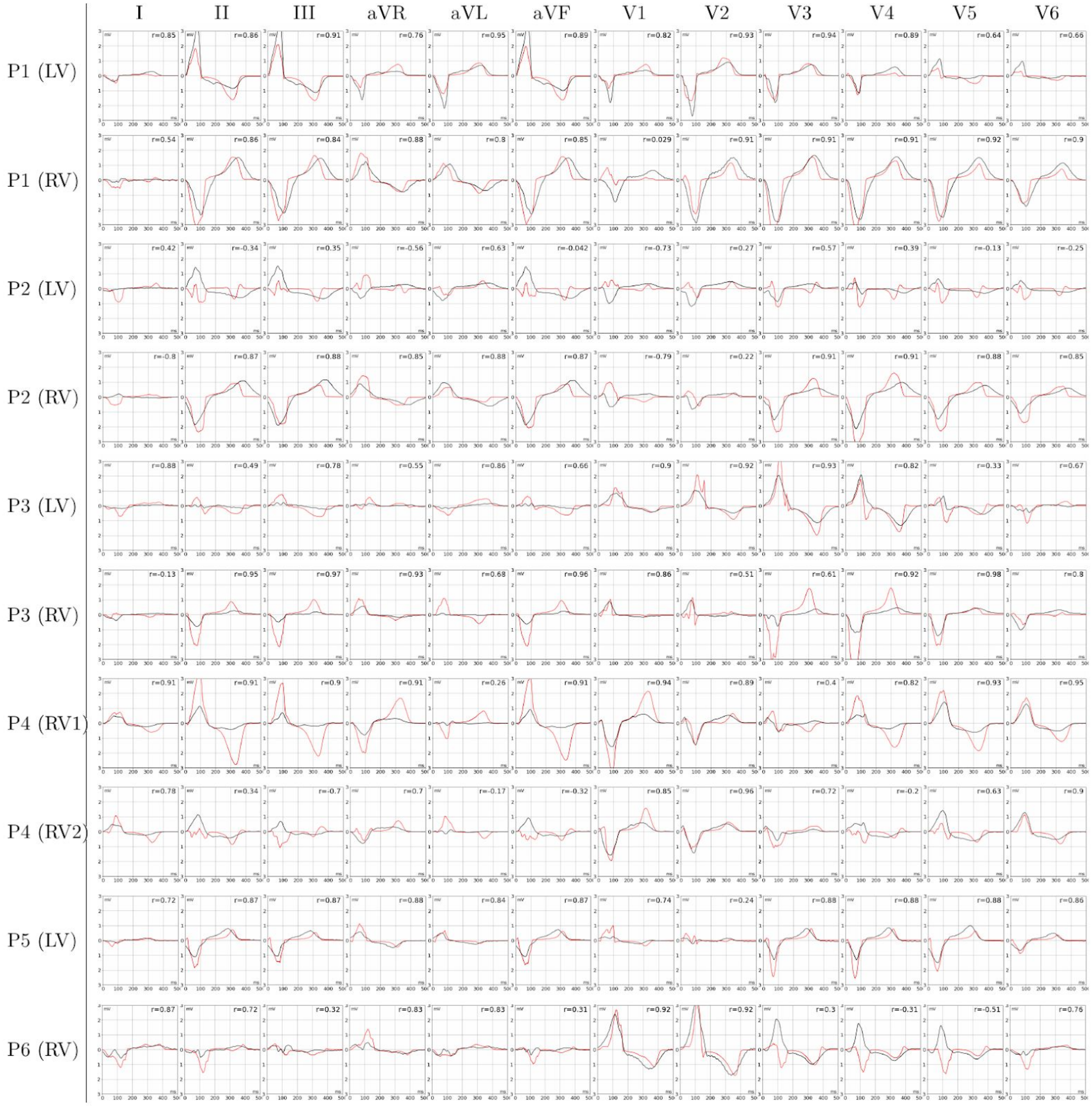

S1 Fig. Advanced visualization for Fig 3 in the main text. This is the comparison of simulated and patient ECG.

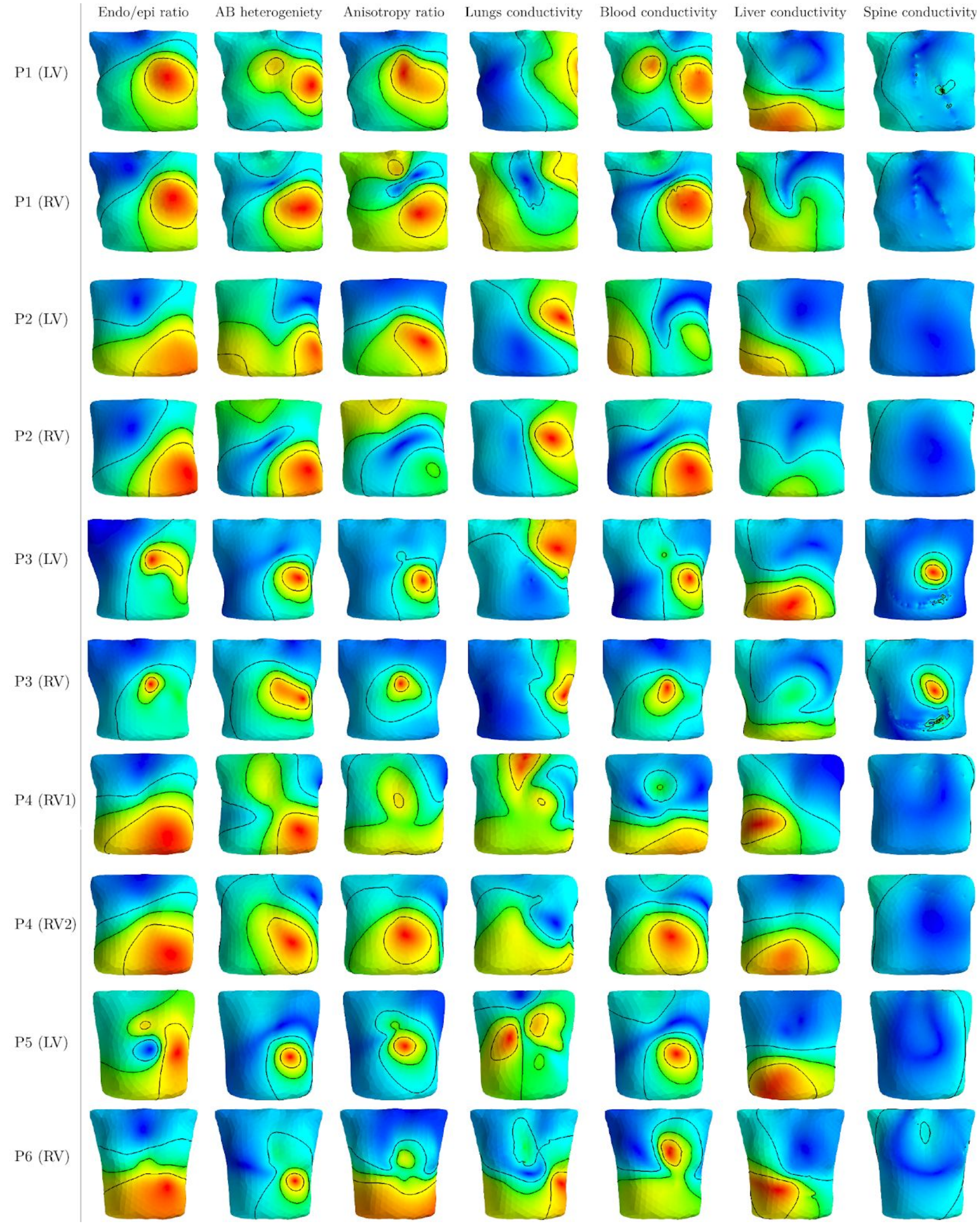

S2 Fig. Advanced visualization for Fig 12 and Fig13 in the main text.

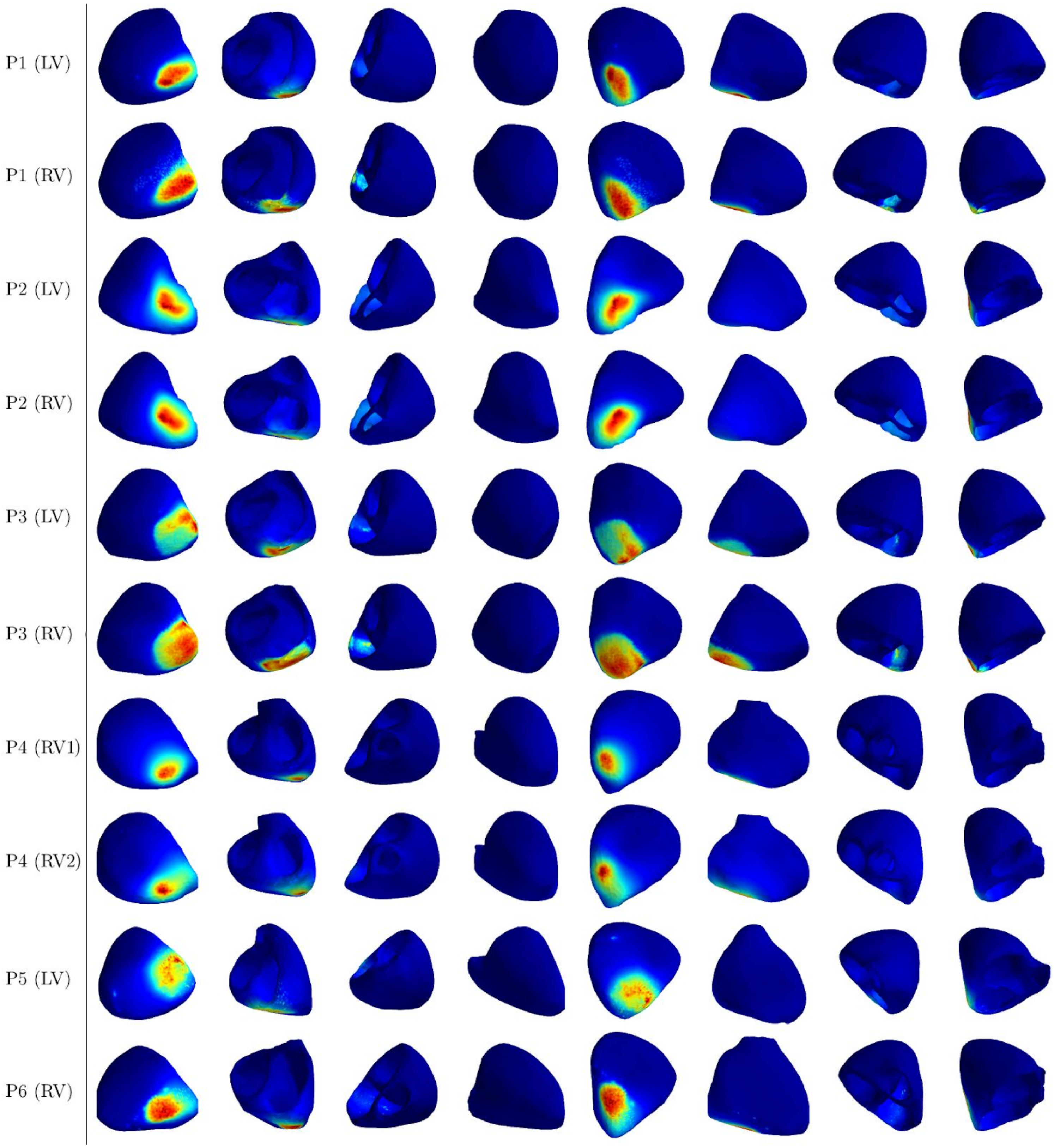

S3 Fig. Advanced visualization for Fig 8 in the main text.

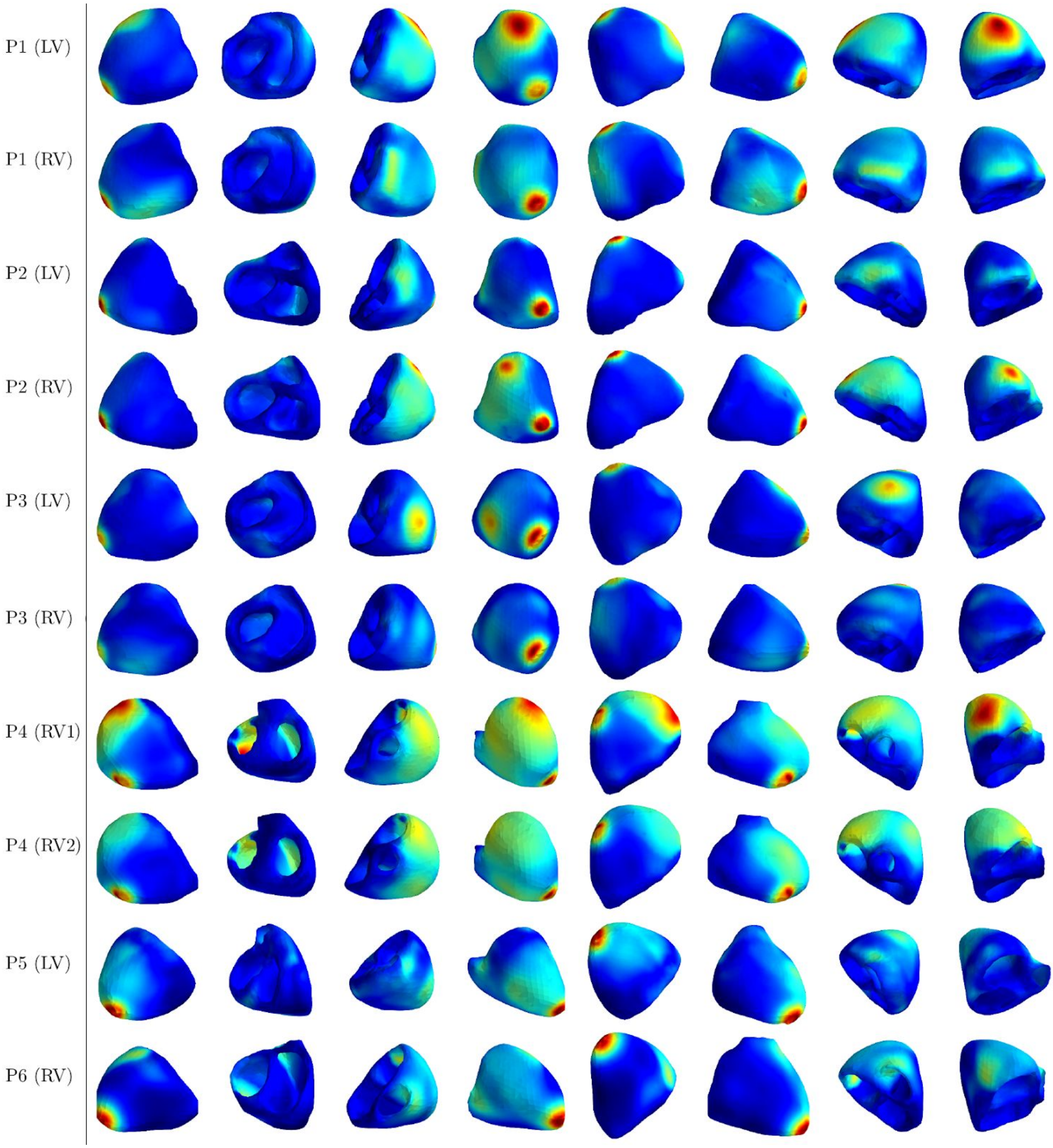

S4 Fig. Advanced visualization for Fig 9 in the main text.

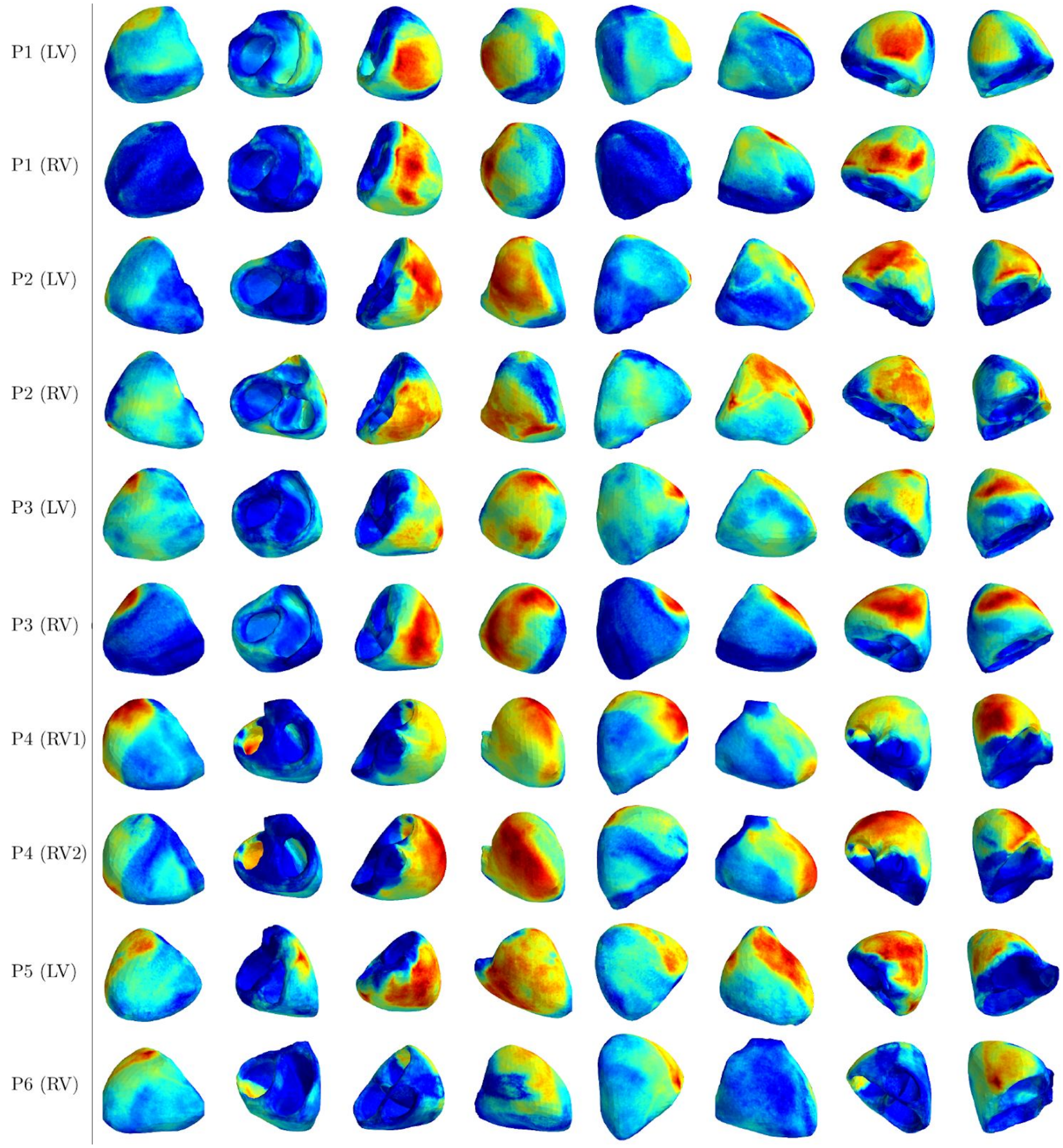

S5 Fig. Advanced visualization for Fig 10 in the main text.

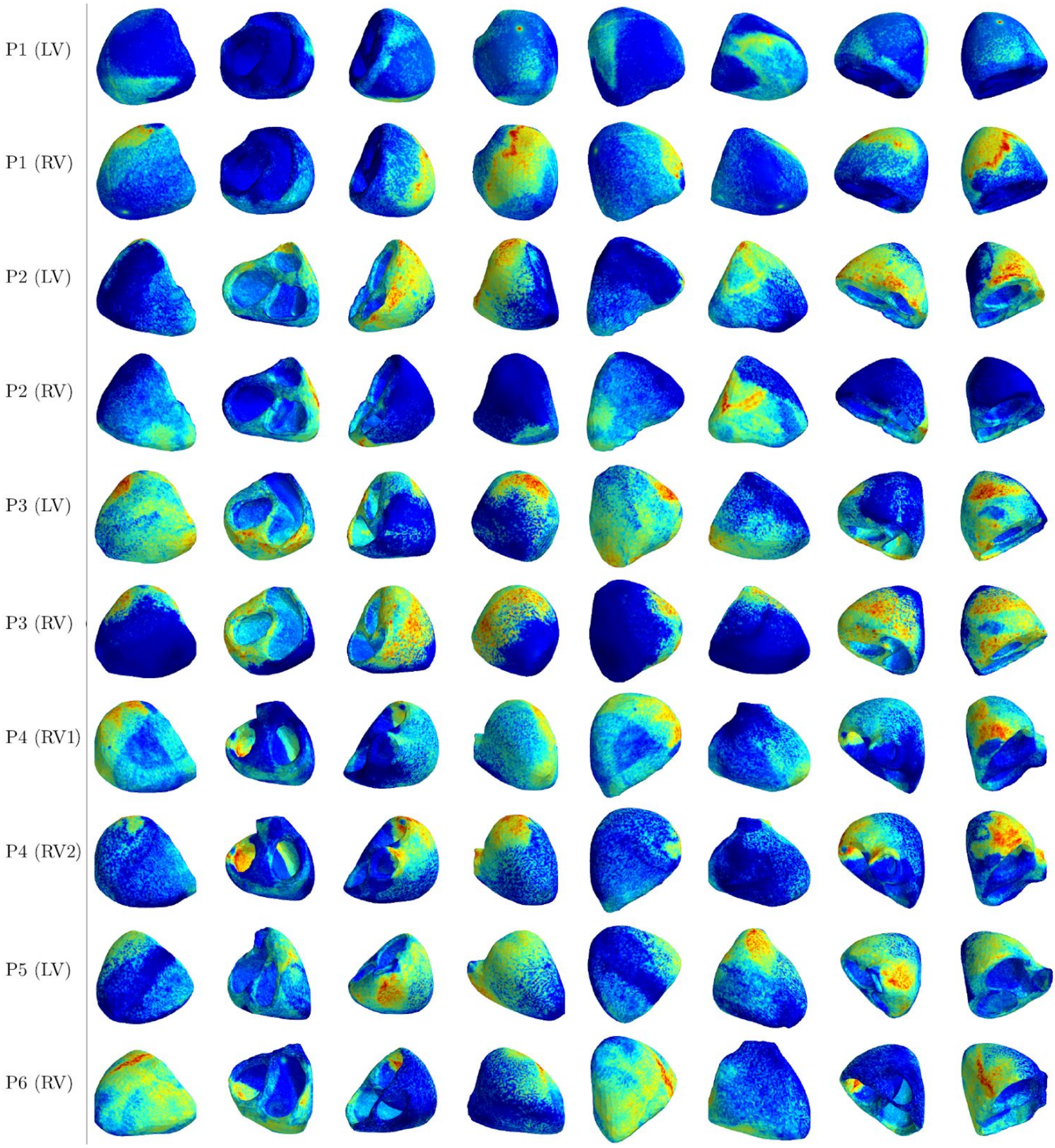

S6 Fig. Advanced visualization for Fig 11 in the main text.
