# Supplementary material for: Parameter variations in personalized electrophysiological models of human heart ventricles
Source: PLoS One. 2021 Apr 28;16(4):e0249062. doi: 10.1371/journal.pone.0249062 (PMC8081243; doi:10.1371/journal.pone.0249062)
Supplement: Attachment — Submitted filename: Response to Reviewers.docx [file pone.0249062.s002.docx]

We thank all reviewers for their meaningful comments. All comments were taken into account and the corresponding changes were made to the text.

***Reviewer #1:*** *This manuscript addresses an important topic, and aims to study how the parameters in a cardiac model influence simulated ECG waveforms. The simulations are informed by data from patients.*

*Although the study is well motivated, I have three major concerns:*

*1. The methodology for sensitivity analysis is not clearly described, and it is unclear how the model parameters have been varied. Table 3 indicates that 7 parameters are involved, and it is not clear that this 7-dimensional parameter space has been explored evenly and completely. At the very least, I would have expected an approach such as latin hypercube sampling to be used. I would recommend that the authors substantially revise their approach in the light of both a recent paper from Saltelli -- https://doi.org/10.1016/j.envsoft.2019.01.012, and a review from the Maastricht group -- https://doi.org/10.1002/cnm.2755. Both of these papers provide definitions of uncertainty and sensitivity analysis. They give a good introduction to methods for sensitivity analysis, and recommend suitable methods for robust and systematic approaches.*

**RESPONSE**: Thank you very much for the review. Replying to the above note, we would like to clarify the approaches we used in this article.

Indeed, we have not used here conventional sensitivity analysis approaches reported in the papers you mentioned. We focused on the different tasks in this study.

First, we compared experimental data recorded in 10 clinical cases for 6 patients at different ventricular activation protocols with simulations computed using the reference model with population-based parameters conventionally used in many modeling studies by different author groups. The model simulations were first compared with experimental data in terms of qualitative correlation metrics. The analysis reveals the different quality of model predictions for different patients and at different ECG lead locations suggesting that model parameters should be tailored to the personal patient data. The main conclusion from this part of the study is that the correlation metrics is a good estimate for the quality of the reference simulations and the identification problem statement. Next point is that even adequate ECG simulation for 12 standard leads cannot guarantee the quality of simulations in the entire body.

The results obtained in the first part of the study raises the question of the justification and adequacy of a parameter identification problem in a physiologically non-implausible range for several chosen parameters. This task was addressed in a simple possible way of analysis using one-by-one parameter variation in the physiological range with fixed other parameters. Here, each free parameter was tested in the physiological range and a relative Euclidean distance (RED) between the simulated and patient ECG was calculated as a function upon the variable parameter value. By plotting such a function, we can see if the minimum for the distance lays within or at the border of the permissive interval. The function visualizes how much the RED changes between the reference and minimum parameter value and how far the minimum is from the reference. Such analysis shows if this parameter variation may essentially contribute to a reduction in the model discrepancy with the experimental data compared to the reference model. The main conclusion from this analysis was that the best parameter values for the minimal RED were more frequently located at the edge of the physiological interval, suggesting more wide permissive intervals for solving the identification problem. We also found a shortlist of parameters essentially contributing to ECG that could be varied to perform model tailoring to experimental data. These parameters were myocardial anisotropy and conductivities of some internal organs.

These predictions were then double-checked in the following analysis of the model sensitivity to variation of the parameters with respect to the reference model. Here, we analyzed if the change in the parameter causes a significant effect on the model simulations as compared to the reference values. The approach for analysis we used in this article was adapted from Keller et.al, 2012 for signal comparison and from Sanchez et. al, 2018 for physiological biomarker comparison. We used integrative (aggregated) relative Euclidean distance between model signals (transmembrane potential, extracellular potential) throughout some myocardial area (ventricular surfaces, entire myocardial volume, regional locations on myocardial surfaces, torso, ECG lead locations) as a measure of the model proximity to the reference outputs. We computed the maximal value of such distances within the physiological intervals for the parameter variation and analyzed if the effect of parameter change is physiologically significant. The main findings of this analysis were that different parameters demonstrate an essential contribution to different model output signals and find 2-3 parameters that variation has the significant impact on either the transmural potential or on the extracellular potential, and on the physiological characteristics of the signals (e.g. amplitudes, durations of ECG complexes, action potential duration). For the first time, we showed regional sensitivity of the myocardial model to parameter variation which then can be used for solving model tailoring tasks.

So, we understand that our uni-parametric model analysis is the first step for further multi-parametric model analysis using combinations of varied parameters in the multi-dimensional parametric space, but we believe that our results allow one to limit the number of free parameters to reduce the complexity of the identification problem.

We have edited the Model analysis section in the manuscript to make clear our approaches and metrics to assess the discrepancy between simulated and experimental data and distinctions between model simulations at varying parameters (see p. 7 in the manuscript). A passage on further application of conventional methods for sensitivity analysis is added into the Limitations (pp. 22) and Discussion (see p. 23. line 668).

*2. The configuration of the ECG is known to depend on activation and recovery times in the myocardium, as well as the torso model parameters. Activation and recovery will be strongly influenced by the model parameters provided in Table 2. These parameters are assigned fixed values based on the literature, but there is no consensus on suitable values for these parameters, with estimates varying by up to an order of magnitude.*

**RESPONSE:** We agree that there is no consensus about a number of model parameters, e.g. surface-to-volume ratio and the membrane capacitance indicated in Table 2. That is why we choose these values from computer modeling that works with good simulation results. We added a notice about that in Methods (p. 8, line 286) and Limitations (p. 27) sections indicating the choice of the parameters used in the study.

*3. Action potential shape also plays an important role in gradients of potential during repolarization, and action potential shape is in turn dependent on the wider set of model parameters. The TNNP model is a reasonable choice for this study, but data from human myocytes support a more gradual repolarization profile (https://doi.org/10.1371/journal.pcbi.1002061), and this may be altered again in patients with heart failure and needing CRT. Furthermore, the ventricles begin to contract during the T wave, which alters the tissue geometry. At the very least, these factors should be considered as significant limitations to the manuscript.*

**RESPONSE:** We agree that the use of different cellular models would additionally support our predictions. OHR2011 is a perfect model that reproduces a lot of experimental data, but this model is more consuming in terms of computational time. For our computationally intense study using the bi-domain model, we chose TNNP2006 as it combines an acceptable accuracy of simulations for cardiomyocyte electrophysiology with the lowest requirements for computational resources.

We agree that accounting for cellular remodeling at heart failure is challenging for future work. The effects of mechanics on the excitation is also a problem which has to be addressed in the future analysis. We added notes on these limitations in the Limitations. (p. 27)

*Minor concerns:*

*There are some issues with language, for example line 133 "structured healthy hearts" should be "structurally", and lines 232 and 234 should refer to "literature" instead of "literary".*

**This was corrected.**

*Figures and Tables need more details in the captions. For example it is not clear what P1..P6 represent (I assume patients), and the colour maps in Figures 2,3,8-13 should be clearly labelled and explained in the figure legends.*

**This was edited.**

***Reviewer #2:*** *The manuscript describes a study in which heart-torso computational models were tested for accuracy and sensitivity to parameters, using electrophysiological data from six patients. I agree that more studies of this type are needed to advance the field of personalized medicine. Your section on metrics was especially well-explained.*

*I have a number of recommendations for improving the manuscript, many of which have to do with clarifying meanings and improving the labeling and descriptions of the figures. My suggestions are described in more detail below.*

**RESPONSE:** We thank the reviewer for valuable concerns on our manuscript.

*1) There are small typos throughout the manuscript. The journal website says “PLOS ONE does not copyedit accepted manuscripts,” so I strongly encourage you to proofread the manuscript very carefully. In addition to some spelling and word-usage errors, there appear to be various LaTeX coding errors. Examples: You should re-check your citations so that none are accidentally duplicated within a list. There are several places where a word like “Sec” or “section” is followed by a blank space, instead of referring me to a specific named section. There are lots of places where upside down exclamation points occur, such as page 13 and elsewhere.*

**This was edited.**

*2) P.2 line 35: “has shortcomings” Please give at least one example of a shortcoming here.*

**RESPONCE:** We edited and clarified this paragraph (p. 2, line 59-69)

*3) Since the introduction is somewhat long, it was hard for me to remember all the points you were making by the time I reached the end of the intro. To help counteract this, on p. 3, line 98, it would be great if you could list a few examples of the most important unresolved questions after the portion that says, “Based on these unresolved questions.” To my understanding, novelty isn’t a requirement for PLOS ONE papers, but if there are novel or unusual aspects to your work, it would be good to emphasize these somewhere inside or near this paragraph.*

**RESPONSE:** We agree with your comments. The Introduction section is essentially rewritten (p. 1-4).

*4) P.5, line 152. “The numbers of used BSM leads are presented in Table 1.” I wasn’t able to find these numbers in Table 1. Which column are they in?*

**RESPONSE:** Sorry about that. We did not show this information in the article. Not more than 6.25% of 224 electrodes were excluded from consideration, because of poor connection with the body. This is mentioned on page 5, lines 171-174.

*5) P.5: In the data preprocessing section, you describe segmentation, mesh choices, and mesh refinement. It would be nice if you could include some commentary (maybe in Discussion or limitations) about how these choices may have impacted your results. I’m not asking you to run more simulations, but it would be good to have some idea of how important you think these segment or mesh choices are.*

**RESPONSE:** We added a new subsection “Reasons of model shortcomings” to the Discussion section (p. 27)

*6) Eq. 1: The first and last lines of Eq 1 are written in a way that it isn’t clear to me how the grad operator should be applied. For instance, in the first line of Eq. 1 I was expecting something more like Grad. ( Sigma_i(Grad V_m + Grad phi_e)) with the extra set of outer parentheses.*

**RESPONSE:** We edited the equations (p. 5, line 202, eq. 1).

*7) P.6, paragraph starting near line 180: Is beta the same as beta_m? Please define phi_b and Sigma_b.*

**This was edited (pp. 6, line 203-216).**

*8) Fig 1 and caption: It would help to add some sentences to explain how the figure was prepared. I don’t understand the sentence “Blue circles present …” I think the word “present” should probably be something else? What are the black dots in the figure? Do the rainbow colors in the boxes mean anything?*

**RESPONSE:** We edited figure caption and legend (p. 10, Fig. 1). The colors of the bars have no meaning. Other marks have been clarified.

*9) Fig 3, Fig 6-12: Please explain color-coding of curves (red vs black or blue vs. green) and/or include a legend.*

**RESPONSE:** Definitions of color-coding in figures are added (see Figs. 1-13).

*10) Fig 4: I can’t see any squares in the plot, only lines with circles. I downloaded the .tif version of the figure and zoomed in and still couldn’t see any squares. Please make your squares and circles larger or more distinctive looking.*

**RESPONSE:** We changed Fig. 4 and the description (p. 14).

*11) P.13, line 440: Is p < 0.03 still for the Mann-Whitney test? If so, you should state this.*

**RESPONSE:** We have mentioned using the Mann-Whitney test where appropriate in the text. We used this non-parametric test because of the small number of observations and the non-Gaussian distribution of the variables.

*12) P.14: Figure 7 should be introduced and described at least briefly somewhere on this page, in addition to just including a caption.*

**RESPONSE:** We have added references to Fig. 6 and 7 in the text (p. 16, lines 707,729).

*13) Fig 8-13: Include explanations in the main text of the paper to say why you’re only showing data from a subset of the patients. For example, please explain why Fig 8 only shows P1-P3. I’m not saying you need to include every patient in every plot, just explain why you showed the ones you showed.*

**RESPONSE:** We showed data from some patient models as representative examples of described spatial patterns of the regional sensitivity (in terms of RED) map on the myocardial surface or torso surface. For the rest of the models, the patterns are similar if not specifically indicated and not shown.

*14) P.15, line 522-524: I can’t tell which of the patients in Fig 11 belong to the “7 of 10” cases. Please list which patients belong to which category.*

**RESPONSE:** We added a list of models with an unstructured pattern. (pp 19, lines 560-562)

Further, regions with a high effect of the endo/epi ratio and blood conductivity variation are co-localized in the late activation zone of the ventricles in 7 of 10 cases except for P4(RV1), P4(RV2) and P5(LV).

*15) P.15, similar to before, Figure 12 should be introduced and described briefly in the text where it first appears.*

**RESPONSE:** We added the description of the Figure (pp 19. lines 565-568). Please, note that the order of Figures 12 and 13 in the new text is reversed.

*16) Style notes: While there is nothing technically wrong with one-sentence paragraphs, I find them strange and wonder (especially in the conclusion) whether you can consolidate your sentences more, though if the one-sentence paragraphs were a deliberate choice that you want to retain, that’s okay.*

**RESPONSE:** We merged one-sentence paragraphs.

*Thanks for the time spent producing the manuscript. I can review a revised version if it becomes available.*

***Reviewer #3:*** *The Abstract begins: “The objectives of this study were to evaluate the accuracy of personalized numerical simulations…” I do not think the results accomplish that objective. For example, “We found a comparatively good correlation (r > 0:72) between the simulated and real ECG for 8 of 10 cases (80%).” First, r > 0.72 seems arbitrary. In Fig. 1 the average r > 0.72 for most data sets, but there is a tremendous variability among recording sites, including one (or many) negative correlations in all 10 datasets. What does this mean? How good an agreement between model and data would be needed for it to be clinically useful? A correlation coefficient may be unbiased, but that is an advantage and a disadvantage: a point-by-point summary of error may or may not highlight relevant features. For example, in Fig. 3 it is noted that the polarity of the QRS complexes are sometimes inverted between data and model; isn’t that a sign that the model is badly wrong? In general, the value of a model depends on how it is to be used, but this is not critically evaluated here. The study does not draw strong conclusions about the value of such personalized models, but the conclusions that are drawn do not seem to be well supported.*

**RESPONSE:** We thank the reviewer for valuable concerns on our manuscript. One of the main objectives of this study was to evaluate the accuracy of personalized numerical simulations of the electrical activity in human ventricles by comparing simulated electrocardiogram (ECG) with real patients’ ECG.

Your major comment on the manuscript was that the results do not accomplish that objective for the following reasons. First, the correlation coefficient between real and reconstructed ECGs seems to be an insufficiently reliable indicator of the quality of modeling accuracy. Second, the article does not critically analyze the results of modeling accuracy and does not make a strong conclusion about the clinical value of this type of model.

We agree with your appraisal, but only partially. The advantages and disadvantages of the correlation coefficient were briefly discussed in the section Methods (p. 8, line 287-297). In particular, we noticed that this metric is weakly sensitive to small changes in signal values and variations in signal amplitudes. However, as we have noted, the correlation coefficient is a generally accepted measure of the qualitative difference between signals and has been used in several studies with a similar design (lines 289-293). The correlation coefficient allows us to compare the quality of our simulation with the published data. This is why we have limited ourselves to providing accurate analysis results in terms of correlation. We choose the level of the mean correlation coefficient above 0.7 as a measure of the strong correlation which is widely agreed in the statistical applications in medicine (Akoglu et.al, 2018). Eight model cases over perform this threshold. Three cases of simulation (P1(LV), P1(RV), and P6(RV)) overperform an 80% level of high accuracy suggested in the other works, e.g. see Keller et. al, 2011. These cases are classified into the first group of high correlation models with CC>0.80 as described in the text (p. 10, line 330).

However, we agree with you that more quantitative and clinically [interpretive](https://context.reverso.net/%D0%BF%D0%B5%D1%80%D0%B5%D0%B2%D0%BE%D0%B4/%D0%B0%D0%BD%D0%B3%D0%BB%D0%B8%D0%B9%D1%81%D0%BA%D0%B8%D0%B9-%D1%80%D1%83%D1%81%D1%81%D0%BA%D0%B8%D0%B9/an+interpretive) estimates of model accuracy would add information content for the results presented in the manuscript. In this regard, we added an analysis using another metric, the normalized mean-square deviation in the revised version of the manuscript. These metrics are also often used to compare bioelectric signals. Accordingly, we added the definition of this metrics to the Methods section and presented the comparison results in terms of this metrics in “Results / Comparison of reference simulations and PECG (p. 10)” section and their discussion in Discussion/ Accuracy of the ECG simulation section (p. 20). These metrics were used in addition to the relative Euclidean distance between simulated and patient data (see the section Model analysis ) which is also a kind of quantitative estimate of the model quality. This was used to assess the possibility to use uni-parametric optimization of the model which allows us to predict the most important parameters affecting the proximity of simulations to the experimental data.

Despite the relatively high average correlation between simulated and real ECGs, which was found in the numerical tests, there was a high level of error for certain models. In the last version of the manuscript, we concluded that the models with the averaged population-based parameter values do not allow us to provide personal ECG modeling for any cases (section “Conclusions”, lines 830 - 836). In the revised version of the manuscript, we further highlighted the negative results about the lack of accuracy and strong variability in the simulation results. In particular, we added data on the variability of the correlation coefficient to the abstract and drew attention to the negative correlation coefficients and the opposite polarity of QRS complexes in some ECG leads in the “Discussion” section. We also noted once again that the clinical application of the models with the averaged population-based values parameter has significant limitations.

*The English is generally OK, with some issues: The sentence beginning on line 53 is awkward. Lines 232, 234 "literature" not "literary" values.*

**This was edited in the text.**
