# Supplementary material for: Parameter variations in personalized electrophysiological models of human heart ventricles
Source: PLoS One. 2021 Apr 28;16(4):e0249062. doi: 10.1371/journal.pone.0249062 (PMC8081243; doi:10.1371/journal.pone.0249062)
Supplement: Attachment — Submitted filename: RESPONSE2.docx [file pone.0249062.s003.docx]

We thank all reviewers for their meaningful comments. All comments were taken into account and the corresponding changes were made to the text.

**Reviewer #1: The revision addresses some of my concerns. However concern 2 in my original review remains.**

**I wrote "2. The configuration of the ECG is known to depend on activation and recovery times in the myocardium, as well as the torso model parameters. Activation and recovery will be strongly influenced by the model parameters provided in Table 2. These parameters are assigned fixed values based on the literature, but there is no consensus on suitable values for these parameters, with estimates varying by up to an order of magnitude." I believe that this point underlies the poor correlations between some of the simulated and recorded ECG signals (e.g correlation coefficients of -1 in Figure 1). I would consider other measures with which to compare the simulated and recorded ECGs: QRS duration, QT interval, Twave symmetry are all metrics that might provide valuable information.**

**I understand the authors' response to my comment about sensitivity analysis. I would like to see a rationale for the analysis of parameters in Table 3. Why include these and not others?**

**RESPONSE:** Your comments show that two points are not clearly stated in the manuscript. The first question is about what the values of the model parameters were used for the reference model and why. The second question is what parameters were selected for variation in the frame of the sensitivity analysis and what was the motivation for this selection. Let us try to give detailed answers to these questions.

The parameters of the model can be divided into three groups. The first group consists of parameters of the cellular model, i.e. parameters of ionic currents in cardiomyocytes. The second group includes parameters of the bidomain model: cell membrane capacity, the surface-to-volume ratio of the cardiomyocytes, and coefficients of the conductivity tensors of myocardial tissue. The third group of model parameters includes parameters of the torso organ conductivities. In addition, each group can be extended with parameters that characterize the spatial heterogeneity of the parameters from this group.

The reference model is a model that we used to compare the simulation results with the ECG of patients and as a reference point for sensitivity analysis. For the reference model, the parameter values were assigned as follows. The parameter values of the first group were taken from the original work (ten Tusscher et. al. 2006), since we assume that TNNP 2006 model of human ventricular cardiomyocytes has an optimal balance of complexity and adequacy. Moreover, we took into account the transmural and apicobasal heterogeneity of the potassium currents (g_Ks, g_to). A significant influence of these parameters on ECG morphology was shown in the work (Keller et. al, 2012).

In our work we used population-based values for the parameters of the second group, based on the previous modeling works (Keller et. al, 2010, Keller et. al, 2012, Boulakia et. al., 2010, Sánchez et. al., 2018), which carefully selected a plausible range of values to simulate adequate characteristics of ECG recorded in patients. In particular, these parameter values allow the model to produce: (1) a realistic conduction velocity 0.5-0.6 m/s in myofiber direction and 0.15-0.25 m/s across the fibers which is reported in literature, (2) to simulate QRS width higher than 100 ms that is close to patient recordings upon the point stimulation.

We understand that these parameters may be patient-specific and vary in different regions of the myocardium, so our assumption should be considered as a limitation of the study. However, variation in the parameters would change the conduction velocity, but not the direction of the wave-front so the choice of the parameters cannot induce wave inversion and essentially improve the quality of simulations in cases where correlations between ECG recordings and simulations are rather poor. In contrast, the local heterogeneity in the conduction velocity can essentially affect the ECG morphology, but this issue was out of the scope of this study.

The third group of parameters included electrical conductivity values of the lungs, blood in the heart cavity and large vessels, liver, and bone tissue of the spine. Thus, all significant electrical conductivity heterogeneities were taken into account in our model with the exception of pericardial fat, ribs, and sternum. We also used population-based values for parameters that have been reported in previous works Keller et. al, 2010, Sánchez et. al., 2018.

When selecting model parameters for variation for the purpose of sensitivity analysis (see Table 3), we focused on those parameters whose effects were not analyzed in detail in previous studies. We intentionally did not analyze the effects of the ionic parameters on simulated ECG as model sensitivity to their variation and effects on ECG characteristics were previously studied in several works in detail (Keller et. al, 2010). However, coefficients of epi/endo and apicobasal heterogeneity was included in the analysis because of the fact that their essential role in the T-wave orientation was previously demonstrated in the model at the normal activation sequence, while no data was available on their significance for ECG morphology in the cases of ectopic action.

It is known that the ratio of cell membrane capacity to the “surface-to-volume ratio” affects strongly the conduction velocity. In our work, we have tried to maintain a realistic conduction velocity for all simulation cases, so we did not vary the membrane capacitance and the surface-to-volume ratio.

When varying the parameters of anisotropic electrical conductivity of the myocardium, we used a special approach. It is known that there is a correlation between the coefficients of anisotropic electrical conductivity of the myocardium. We used Roth et. al. mathematical framework that establishes a relationship between extracellular and intracellular conductivities. This approach allows us to vary the values of the four electrical conductivity parameters, based on varying the value of only one parameter, the so-called anisotropy ratio ($\lambda_L/\lambda_T$). We used a variation of this parameter instead of variations of the four parameters for our sensitivity analysis.

Finally, we varied the conductivity of the internal organs and blood intending, first of all, to study the regional effects of these variations. In particular, we hypothesized the regional effects of variations of spine conductivity on the ECG characteristics. However, modeling results did not support this hypothesis as discussed in the paper.

Thanks to your comments, we have seen that these important points are not clearly stated in the manuscript. In particular, the caption to table two contains an incorrect statement.

We have made appropriate corrections in the "methods" and "discussion" sections and in the Tables 2 and 3.

Answering your suggestion on using ECG features to estimate the effects of parameter variation, we provided such analysis as reported in the Section “Effects of parameter variation on properties of myocardial depolarization and repolarization”. We analyzed the effects of parameter variations on the amplitude and time-to-peak values of QRS complexes and T-waves, which can be accurately detected by ECG signals. However, we did not use such parameters as QT-interval, QRS duration, J-point, T-wave asymmetry when comparing the simulation results with the patients' ECGs. We understand these ECG characteristics are often used in clinical practice. The problem is that their clinical value was investigated only for sinus rhythm. Their applicability in the case of ectopic heartbeats originated from various sites of the ventricles is not clear. Moreover, detecting T-wave boundaries and measuring the QT-interval is challenging for ectopic ventricular contractions.

**There remain some problems with the English, and the manuscript must be checked by a native English speaker.**

**For example:**

**Line 265 'propose' -> 'purpose'**

**Line 286 'includes' -> 'include'**

**Line 293 'metrics is' -> 'metric is' or 'metrics are'**

**Table 4 'electode' -> 'electrode'**

**RESPONSE:** All noted mistakes were fixed. Additionally, we used an external proofreading service to fix as many mistakes as possible.

**Reviewer #2: Thank you for revising the manuscript and for your responses to my comments. I have a major comment and a minor comment about the revised version.**

**Major comment: My main remaining issue is that my previous advice (to proofread the manuscript very carefully) wasn’t addressed as thoroughly as I’d hoped. I appreciate that the authors fixed typos that I explicitly pointed out, but what I was trying to convey in my first review is that there were lots of other mistakes that I didn’t have time to list individually. Although I understand that some typos are inevitable in a manuscript of this length, the number of mistakes is still higher than what I’m used to seeing. I encourage the authors to find a native English speaker (or as close as they can find to one) to read the manuscript carefully, though some other proofreading approach would be needed to handle issues with figures and equations. Here are some examples of problems I encountered:**

**RESPONSE**: We apologize for the quality of the previous text. We have used a proofreading service for the current version.

**- I can’t read the numbers along the axes in certain figures. For example, the axis numbers for the blue and green ECG curve plots in Figures 9-11 are especially hard to read. The figures are very small and blurry. Downloading the .tif files didn’t help clarify anything. Perhaps the figures look good on your side, but I can’t see them clearly. Were you able to download the version supplied to the publisher and check all figures for clarity?**

**RESPONSE**: We have improved quality of images for Figures 3,4, 9-11.

**- There are typos or formatting errors in several of the equations that were added to the revised version, including but not limited to Eq. 11.**

**RESPONSE:** Typos in Equation 11 and some equations in the text were fixed.

**- There are various grammatical and word-usage errors. For example, there are cases where a word is spelled correctly but the wrong word is being used, such as in the caption of Figure 4.**

**RESPONSE:** We have fixed word-usage errors in the caption of Figure 4,5 and some other places in the text.

**There are many other mistakes besides the ones I pointed out above. I don’t think it should be my role to fully edit the manuscript, and I don’t think PLoS-ONE will do it, so I’m not sure how to proceed. If the journal’s policy is that they don’t provide copy-editing, that puts more of the proofreading burden on the authors compared with some other journals.**

**RESPONSE:** We used an external proofreading service to fix as many mistakes as possible.

**Minor comment: Please clarify what types of mesh nodes are included in set “I” for Eqs. 12, 13, 15. I can find an explanation of what “I” is where Equation 14 is introduced but I don’t know if that description of “I” pertains to the other equations as well. It’s possible this was addressed somewhere and I missed it. It would be fine to define the sets later when relevant tables or figures are introduced in Results, if that is easier.**

**RESPONSE:** Done.

**Reviewer #3: The authors have addressed my concerns. However, I remain unsure about how such personalized models would be used clinically, and what level of agreement between model and data is needed for the model to be useful.**

**RESPONSE:** A brief overview of possible clinical applications of personal computer models of cardiac electrical activity is given in the Introduction section of this manuscript. In particular, personal models can be useful in planning catheter ablation of cardiac arrhythmias, optimizing cardiac resynchronization therapy, and stratifying the risk of sudden cardiac death in patients with ischemic and non-ischemic cardiomyopathies, etc. (see references [2]-[8]).

What level of agreement between model output and data is needed for the model to be useful in clinics? Theoretically, modeling errors should not exceed the typical ECG recording error (electrical and muscle noise, changes in the contact resistance of the ECG electrode with the skin, inaccuracies in determining the positions of the standard ECG leads (V1-V6), etc.) and the level of ECG variability caused, for example, by the patient's breathing. However, each medical application requires its own level of modeling accuracy, which must be identified through clinical research. Nevertheless, we may assume that 4 out of 10 cases of our simulation provided accuracy close to that required for clinical applications. On the one hand, this shows that the required level of model accuracy is fundamentally achievable. On the other hand, modeling using averaged population-based parameters does not work well and this approach cannot be directly translated into clinical practice.

We have added a brief comment on the required modeling accuracy for clinical applications in the discussion section of this manuscript (page 24, Accuracy of the ECG simulation).
