# Supplementary material for: Parameter variations in personalized electrophysiological models of human heart ventricles
Source: PLoS One. 2021 Apr 28;16(4):e0249062. doi: 10.1371/journal.pone.0249062 (PMC8081243; doi:10.1371/journal.pone.0249062)
Supplement: Attachment — Submitted filename: Responce3(1).docx [file pone.0249062.s004.docx]

We thank the reviewer for meaningful comments and especially for attention to technical details and errors in the figures of the manuscript.

Reviewer #2: Thank you for addressing my comments. I appreciate that you hired a proofreading service and that you boosted font sizes on certain figures.

However, the level of care taken in checking the figures still falls short of what I was expecting, since it appears that new figure-related mistakes were introduced in this draft, and in at least one case a mistake persisted from the previous draft. Steps that I think your team (or the proofreading service) should always carry out when submitting a manuscript are to (1) compare every figure caption to the version of the figure that you uploaded. Are the number of elements described in the caption the same as the number shown in the figure? (2) Compare every figure with its counterpart from the previous draft. If there are any significant changes to the figure format (aside from just editing for clarity), determine whether these changes were intentional, and if so, explain the rationale for the changes in your response to the reviewers.

I’d prefer to focus my efforts on reviewing technical content rather than manuscript format. If for some reason I’m the one who’s looking at the wrong files, I apologize, but otherwise I find it hard to build trust in the rest of the manuscript, or to determine whether the figures support the conclusions, if the steps I described previously aren’t followed consistently.

Unfortunately, we did not notice some errors in the final version of the manuscript, which was automatically generated by the journal's submission system, and we are sorry for that. In particular, Figure 1 was not replaced and remained the same as in the previous version. The same image was used for Figures 12 and 13. These errors occurred at the stage of uploading the manuscript on the journal's website.

We carefully checked all the drawings and figure captions in the automatically generated document and made the necessary corrections.

Furthermore, we once again checked all the numerical values in the text, tables, and figure captions. We fixed the numerical values on page 12 (lines 411-419) and page 15 (lines 512-529). We also presented the numerical data on pages 16 (lines 546-558) in a ‘mean+std’ format. A caption in Figure 3 was corrected according to the presented figure.

We once again used a proofreading service and corrected the grammar, spelling, and punctuation of the text as suggested by the editor.
